# Sustainable evaluation procedure for residual commingled waste after recycling, factoring in the percentage content of solid components

**DOI:** 10.1371/journal.pone.0345320

**Published:** 2026-04-01

**Authors:** Zbigniew Jelonek, Iwona Jelonek, Przemysław Rompalski, Justyna Guzy-Proc

**Affiliations:** 1 University of Silesia in Katowice, Faculty of Natural Sciences, Institute of Earth Sciences, Sosnowiec, Poland; 2 Centre for Biomass Energy Research and Education, University of Silesia in Katowice, Sosnowiec, Poland; 3 Central Mining Institute – National Research Institute, Department of Solid Fuels Quality Assessment, Katowice, Poland; St. Pius X College, Rajapuram, Kasaragod-Kannur University, INDIA

## Abstract

The management of residual municipal waste after recycling is becoming an increasingly problematic procedure, while the challenges related to this process are multi-dimensional and encompass environmental, economic and social factors. As per the recommendations of the European Union, it is more beneficial for the residual fraction to undergo disposal via waste-to-energy conversion in specialist incineration plants and monitored low-power boilers than by landfilling. Such a process not only limits the negative impact on the environment that is characteristic of landfilling but also makes it possible to obtain additional electric and thermal energy. In this paper, the authors indicate the key role of the precise determination of solid waste composition before thermal conversion or storage. They also present a new optical method of residual fraction analysis for solid component proportion determination. It should be stressed that the precise knowledge of the waste composition can streamline decision making as regards selective waste recycling and reprocessing, including, e.g., excess organic matter separation. The conducted analyses showed that with an increase in the organic fraction content in waste from 30% to 90%, there is an increase in the emission of, among other things, particulate matter from 124.96 mg/m^3^ to 393.47 mg/m^3^, CO from 819.06 mg/m^3^ to 2161.67 mg/m^3^, and VOCs from 0 mg/m^3^ to 117.1 mg/m^3^.

## 1. Introduction

The management of waste, including its storage, thermal disposal and recycling, is of key significance in the context of global challenges such as climate change or soil, water and air contamination [1]. Furthermore, the responsible use of raw materials and waste is necessary to achieve sustainable development [2,3]. Municipal waste constitutes a problem both in terms of its disposal facilities’ aesthetics, as well as its storage [4]. It contributes to the emissions of noxious odours and greenhouse gases and occupies significant areas of land, which limits the options for its alternative use [5–7]. Effective waste management, including recycling and sorting process improvements, has become one of the goals in the UN 2030 Agenda for Sustainable Development. Some of the action as part of the Agenda is carried out towards greenhouse gas emission limitation and natural resource saving. As regards waste, the adopted goals are to be achieved by processes of municipal waste conversion and repurposing [8]. Currently, advanced recycling finds common application in countries with high standards of living, and it constitutes a key element of sustainable waste management [9]. It is also becoming the basic element of waste management in conscious systems of environmental protection [10]. Nevertheless, the full recycling of useful materials in 100% constitutes an enormous challenge at the current state of technology and is impossible in many cases. It should however be stressed that some residual fraction is always left even when the best waste conversion and recycling systems are applied [11], and even a minor percentage of this fraction, when considering the global household waste production, can pose a significant problem to the environment when such residues are to be stored. As some materials are hard to recover due to their composition or physicochemical state, not all waste can be effectively processed and repurposed. These include fragments of mixed-material waste or fine elements from electronic waste as well as poorly separable composites and materials that have lost their recycling properties as a result of prior processing cycles [12,13]. The residues are analysed for their potential application as a material in energy processes, e.g., in waste incineration plants, where they can be used for producing thermal or electric energy. The incineration of the residual fraction requires maintaining conformity with rigorous emission standards as well as applying advanced flue gas purification methods and ensuring appropriate storage before the process (Directive 2010/75/EU). The storage before incineration should be short-term, and the dedicated storage sites should be correctly secured to properly protect the soil from potential contamination, including leaching from the stored waste [14,15]. However, from the perspective of environmental benefits, the thermal conversion of the residual fraction appears to be much more advantageous than landfilling in specifically designated sites. Long-term storage involves the occupation of major areas for waste dumps, which become irreversibly lost as regards their potential future repurposing. Landfilled materials must also be carefully secured to prevent soil contamination as well as the emissions of greenhouse gases and unpleasant smells [16]. Measures must be taken to minimise the risks related to the self-heating and ignition of the stored waste as well [17,18]. Furthermore, in the majority of cases, the natural disposal of such materials lasts hundreds of years, which involves the necessity to install additional facilities and incur the costs related to monitoring the waste dumps [19–21]. Meanwhile, after thermal conversion, the residual waste not only generates additional energy but also undergoes a significant reduction in volume by changing into ash. In this way, the obtained ash can be used as a fertiliser (should its physicochemical analyses yield positive results), while in extreme cases of ash exhibiting higher pollutant concentrations, it can be used as an additive in the building materials industry [22,23].

In Europe, the level of municipal waste processing is varied and depends on numerous factors, including the environmental policy, infrastructure and social awareness. Eurostat data [24] reveals that municipal waste production is very diverse in individual EU member states. In 2022, the most municipal waste was produced by the citizens of Austria (827 kg per capita), Denmark (787 kg) and Luxembourg (720 kg). The least was produced in Romania (301 kg), Poland (364 kg) and Estonia (373 kg). The recycling levels in the individual EU states vary greatly, ranging from 6% to 83%, which indicates the disproportions in the approach to waste management. Outside Europe, the level of municipal waste processing is very diverse as well, and likewise depends on numerous factors, such as economic development, infrastructure, social environmental awareness and legal regulations. For example, in developed countries, such as the United States, the recycling level is very low, at 32%, and it is even lower in Japan, at barely 20%. In developing countries, such as India [25] or Brazil, the level of waste processing is lower still, and the problem often stems from the lack of necessary infrastructure and low environmental awareness. In many of these countries, the waste is often stored in waste dumps or incinerated in inadequate facilities [26], which leads to negative impact for the environment and human health. Despite many hazards, in the global context the thermal conversion of waste is acknowledged as one of the most responsible and pro-environmental solutions for the problem of residual waste [27,28]. Following the example of Sweden [29], where recycling and energy repurposing are the pillars of the waste management system, other global economies should abandon waste storage both on land and in oceans. That is because waste storage is a solution much less advantageous from the economic and environmental perspective compared to waste-to-energy conversion, as can be seen in the energy repurposing applications of waste in Nordic countries [30,31].

### Materials

**Fig 1:**
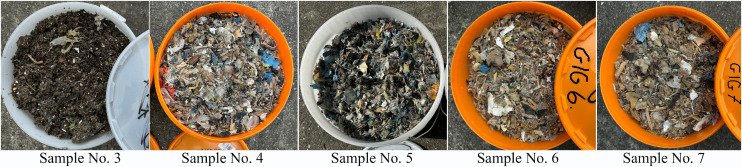
Representative samples of waste collected from their storage locations.

The object of study included the residual fraction from municipal waste material recycling processes. The waste was collected from 5 facilities specialising in the sorting and mechanical and biological conversion of waste, found in various regions of Poland. The test material was collected from piles formed where the residual fraction obtained after recycling was stored. In each of the 5 sampling sites, the waste in the piles was pre-mixed manually by means of shovels at the sampling spot, to a depth of about 1 m. Afterwards, the thus mixed waste was separated from the rest of the pile by forming a mound with about 100 kg of the mixed material, which was then subjected to quartering until a sample with a weight of about 4 kg was obtained. The samples were secured against contamination by placing each in one of 5 separate containers (Fig 1).

## 2. Methods

Microscopic studies of the residual waste samples were performed to determine the proportions of solid fractions: organic matter (modern biomass, woody biomass, plant- and animal-based organic household waste, cellulose) to fossil and/or processed organic matter (wood tar, coal, coke) and petroleum products (plastics, rubber, paint, grease, glue, polymer resin, tar, liquid petroleum products) as well as inorganic matter (sand, quartz, soil, stone powder, ceramics, glass, metal, rust, ash, slag). The applied petrographic analysis consisted in the optical assessment of preparations based on the tested material in reflected light using oil immersion per the methodology developed for charcoal, coal and biomass briquettes, and pellets formed from organic and inorganic waste [32,33]. To perform the microscopic analyses, microsections were prepared from the sampled material with a mass of about 5 kg, in the following sequence: If necessary, the material is first dried in room temperature to a humidity of ≤ 15%. Afterwards, the entire sample is ground to a grain size of 0.5 to 1 mm. The ground material is subjected to quartering until a sample of about 0.5 kg is obtained. To eliminate grains of under 0.5 mm from the material, the sample is screened through sieves with a mesh size of 0.5 to 1 mm. About 6 g of the sample are collected from the sieve with a mesh size of 0.5 mm. The microsections are prepared in sequence from 6 g of waste with a grain size of < 1.0 mm collected from the 0.5 mm sieve, thoroughly mixed with synthetic resin with a hardening agent (cold mounting resins). The mixed sample is formed in a 4 mm-diameter cylinder and left until the resin sets. Afterwards, the sample is subjected to manual or automatic polishing on one side in a grinding and polishing machine with P-800 grit wet sandpaper. The polishing is performed for 1–2 min during the axial rotation of the wet disc with the microsections. This is repeated two more times, where first the sandpaper is changed to P-1200 grit and finally to P-4000. After the final polishing, the polished surface is thoroughly rinsed with water. At the end of the process, the obtained microsection is placed in an ultrasonic cleaner filled with distilled water for 5–10 min in order to remove any potential impurities remaining after the polishing. After the microsection is rinsed, it is left for about 6 hours in room temperature to allow the excess moisture to evaporate before the petrographic analysis can be commenced. The analysis consists in identifying the individual components or their groups on the polished surface of the microsection in 500–1000 points determined by the intersection of the cross wire in the microscope ocular. An ocular magnification of 10x and objective magnification of 50x is applied. Before the analysis begins, the microsection is fixed on a microscopic slide on plasticine, after which it is levelled by means of a mini-press and placed in the grip of the object stage mounted on the microscope frame. After a solid component is identified, it is registered in dedicated petrographic analysis software using a keyboard or other instrument, and the image in the field of view is always moved automatically by the same distance. Should the cross wire intersection in the ocular fall on the binding resin, the identification is not registered, and the image is only moved to allow continuous observation. The analysis enables component identification and the qualitative and quantitative determination of individual waste component contents, encompassing both compact and loose solids.

The elemental composition of the samples was determined by non-destructive energy dispersive X-ray fluorescence (XRF). The measurements for the individual samples were conducted in situ during their sampling by means of the Vanta portable XRF spectrometer. Each sample was individually placed in the Vanta measuring station in dedicated Olympus containers. During the measurements, the samples were subjected to automated analysis using the algorithm of basic parameters measured by means of three scanning beams [34,35].

The physicochemical tests determining the sample moisture, ash content, combustion heat, calorific value, total sulphur, chlorine and mercury were performed in an accredited analytical laboratory (results in the repository). Additionally, to determine the organic matter content and ensure repeatability, apart from optical methods, chemical methods per standard PN-EN ISO 21644:2021-07 [36] were applied as well.

**Fig 2:**
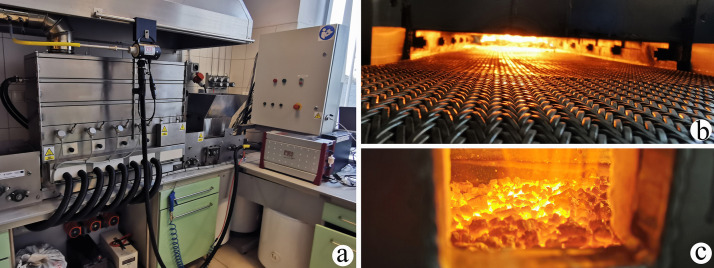
Measurement stand with the integrated experimental stoker-fired boiler, a. laboratory boiler and measurement stand, b. view of the furnace from the ash bin, c. side view of the furnace.

The waste incineration process was conducted in a stoker-fired laboratory boiler with a travelling grate designed for solid fuel combustion under controlled conditions (Fig 2). The device with a thermal power of up to 25 kW is equipped with control systems and software enabling the recording of process parameters such as flue gas temperature, temperatures under the furnace roof and temperatures over the fuel bed. The pressure measurements and particulate matter extraction were carried out in existing gauging sections by means of the ZAM Kęty P10-ZA automatic gravimetric dust sampler and an aspiration probe with an operating temperature range of up to 500°C, with an aspiration head appropriate for the gas velocity and a measuring filter with filtration membranes. The particulate matter samples were weighed in the laboratory on WA-34 scales to determine the mass of the settled dust. Particulate matter concentration measuring range in the channel: (0.001–100) g/m3. Differential pressure measuring range Δpv ± (0 ÷ 1500) Pa. The gaseous component emission measurements (CO_2_, O_2_) were carried out based on standard PN-ISO 10396:2001. The Horiba PG-350E EDR gas analyser was used for the measurements, and the device was calibrated before testing using Air Liquide and Siad calibration gases. The total organic volatile carbon (TOVC) measurements were conducted based on standard PN-EN 12619:2013-05 [37], and they were performed by means of the LAT volatile organic compound analyser with a measuring range of up to 2000.0 mg/m^3^. The atmospheric conditions on the day of measurement were determined by means of a thermo-hygro gauge and the E + E Elektronik Omniport 30 digital meter. The gas temperature in the channel was measured by the SE 520 digital thermometer with a type K thermocouple with a measuring range of up to 600°C and a resolution of 0.1°C. The relative flue gas pressure measurements in the channel were carried out using the MC-5–3 electronic differential pressure gauge with a measuring range of up to 5 kPa. The flue gas velocity and flow in the channels were determined by control measurements in the auxiliary system of the P10-ZA 2000 dust sampler using a velocity probe – type S impact pressure tube and pressure gauges in the dust sampler’s central unit. The flue gas moisture measurements were performed in the auxiliary system of the P-10 ZA 2000 dust sampler using the ZPW-10 psychrometer with dry and wet thermometric resistive sensors.

The applied analysis methods are generally available and commonly used; they do not require permits or licenses.

## 3. Results

Two analytical methods were applied to determine the percentage organic matter content in the tested waste samples. The chemical method was used to identify the proportion of the organic matter to the inorganic matter, while the petrographic method made it possible to not only determine the organic content in the tested samples but also to measure the percentage contribution of the remaining solid components in the material. It should be noted that the residual fraction remaining after the sorting and preliminary recycling of household waste is often designated for landfilling or energy recovery. Petrographic analysis enables the identification and quantitative determination of waste components (mineral, organic, petrochemical matter) and the precise isolation of solid components that are difficult to identify. It is particularly useful where other methods (e.g., sieve or morphological analysis) fail [38]. Beyond providing information on combustion conditions and emissions depending on the RDF (Refuse-Derived Fuel) composition, petrography can also be used to evaluate the effectiveness of sorting in removing materials like glass, metals, plastics, and other solid materials from the waste. The presence of too high a proportion of secondary raw materials in the residual fraction may indicate the need to adjust the sorting parameters. Furthermore, this analysis allows for the microscopic analysis of agglomerates and contaminants, identifying their genesis, e.g., organic materials cemented with mineral, petrochemical, or metallic materials, or difficult-to-separate composites [39]. Despite its potential, the application of this technique in the industrial scale of waste management may encounter certain barriers in interpreting the results. Municipal waste and its residual fractions are extremely heterogeneous, so obtaining a representative laboratory sample, especially from multi-layered piles, requires careful and sometimes multi-stage collection of the test material.

The analysis results are compiled in Table 1, part A, lines 1–7, where the first line presents data from the organic matter content analysis in the tested waste as performed in an accredited laboratory. The data in lines 1–2 was used to compare the organic matter content in the samples using both the methods, and thereby to evaluate the conformity of the obtained results. The remaining components and their percentage contributions as determined by way of petrographic analysis are available in lines 3–7. The individual element content in the tested material as obtained by way of XRF analysis in the field is presented in Table 1, part B. The results of physicochemical analyses performed at the laboratory are provided in Table 1, part C, with determination of the following parameters in the tested samples: sulphur, chlorine, mercury, calcium oxide, humidity, ash, combustion heat and calorific value. The average operating parameters during the sample incineration are presented at the end of Table 1, in part D.

**Table 1:** Compilation of the tested residual waste sample analysis results. A – petrographic analysis results. B – elemental content results obtained by XRF. C – physicochemical analysis results. D – analysed sample incineration results.

A										
No.	Analytical method	Component	Sample No.							
			5	4	6	7	3			
1	ISO 21644:2021–07	Organic matter (bio-waste)	29.6	39.3	62.7	86.2	91.3			
2	Petrographic analysis	Organic matter (bio-waste)	28.4	38.8	63.6	85.6	90.7			
3	Petrographic analysis	Inorganic matter	44.8	35	16.6	6	2.2			
4	Petrographic analysis	Petroleum products	25.9	25.9	19	7.8	7			
5	Petrographic analysis	Coal	0.4	0	0	0	0			
6	Petrographic analysis	Charcoal	0.5	0.3	0.6	0.4	0.1			
7	Petrographic analysis	Other	0	0	0.2	0.2	0			
Total (lines 2–6)	100%	100%	100%	100%	100%					
B										
**Elements**	**Sample No.**									
	**5 ppm**	**4 ppm**	**6 ppm**	**7 ppm**	**3 ppm**					
Al	990	0	1650	5270	4500					
As	4	12	0	9	4					
Ba	132	159	207	102	308					
Cd	0	28	39	0	0					
Cu	75	201	94	92	74					
Fe_2_O_3_	1780	1510	2740	6320	6470					
K	580	1341	1921	2613	7530					
La	108	107	162	138	263					
Mg	0	1090	800	4800	5700					
Mn	582	526	475	1490	1060					
Mo	2830	740	2020	1000	1500					
Nd	327	182	549	591	780					
Ni	20	0	0	30	42					
P	116	384	421	1870	2060					
Pb	30	93	38	77	29					
Pr	204	123	427	214	736					
Si	8040	2628	7420	5680	9010					
Ti	1360	3180	3220	5200	2140					
U	37	29	15	11	0					
Zn	412	370	1173	585	260					
C										
	**Parameters**				**Elements**					
**Sample No.**	**Sample humidity**	**Ash**	**Combustion heat**	**Calorific value**	**S**	**CaO**	**Cl**	**Hg**		
	%	%	J/kg	J/kg	%	%	%	mg/kg		
5	14.2	29.6	16744	15609	0.19	1.78	0.28	0.12		
4	17.5	15.8	21910	20160	0.19	1.95	0.25	0.12		
6	12.7	11.6	21050	19320	0.18	3.45	0.41	0.14		
7	24.7	15.6	16750	15210	0.1	3.22	0.74	0.1		
3	27.2	16.8	14550	12900	0.15	3.09	0.75	0.16		
D										
Parameters										
**S.No.**	**mg/m** ^**3**^	**mg/m** ^**3**^	**mg/m** ^**3**^	**mg/m** ^**3**^	**mg/m** ^**3**^	**%**	**%**	**°C**	**KW**	**°C**
	**Dust**	**SO**_**2**_	**NOx**	**CO**	**VOC**	**O**_**2**_	**CO**_**2**_	**Flue gas temp.**	**Power**	**Furnace temp.**
5	124.96	27.74	76.16	819.06	0	17.5	2.97	234	14	687
4	218.81	14.39	78.8	801.9	65.35	17.5	2.97	210	14	666
6	344.78	27.06	66.43	1340.75	45.1	17.6	2.85	221	13	665
7	391.98	26.31	48.09	1627.85	94.5	17.5	2.4	186	14	664
3	393.47	31.88	81.56	2161.67	117.1	17	3.2	154	15	688

## 4. Discussion

**Fig 3:**
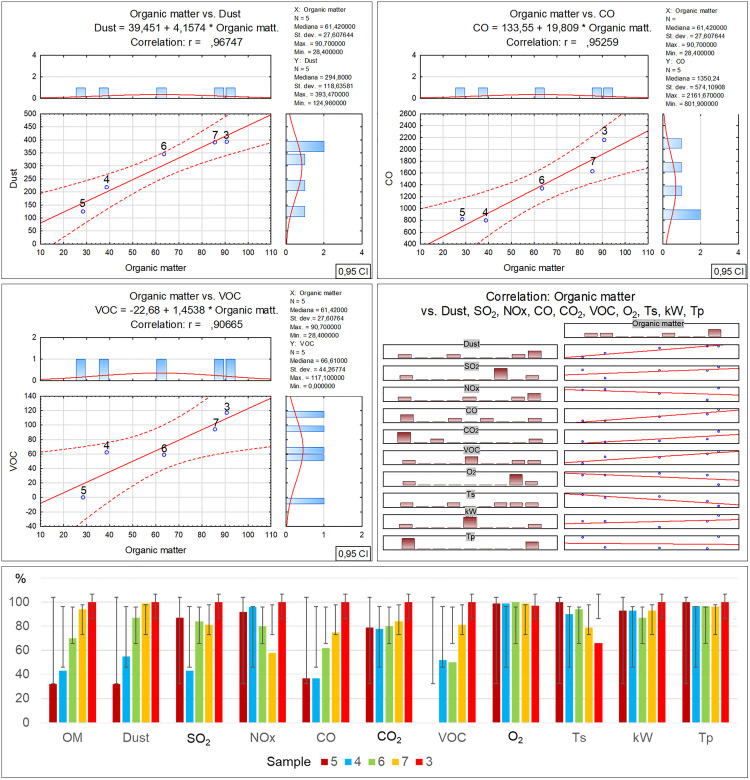
Correlation and regression analysis between organic matter content and various incineration parameters for residual waste fraction samples.

**Fig 4:**
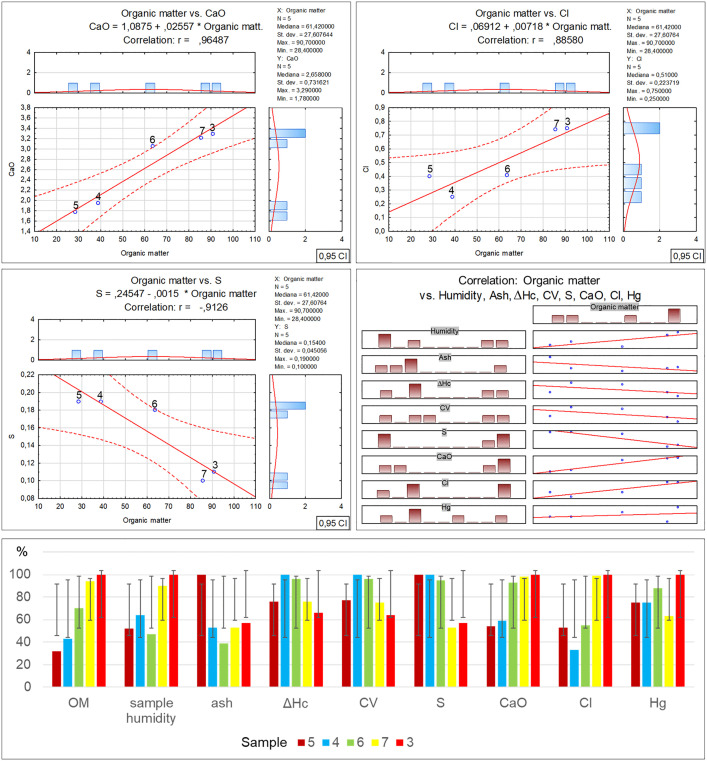
Correlation and regression analysis between organic matter content and chemical/fuel properties in residual waste fraction samples.

**Fig 5:**
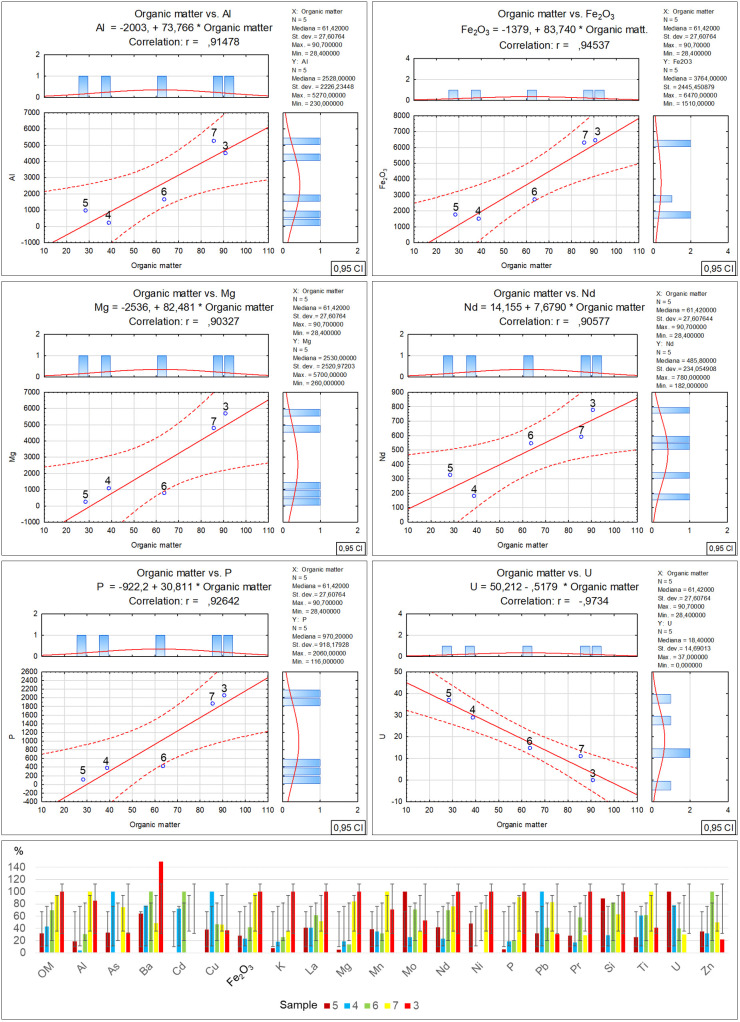
Correlation and regression analysis between organic matter content and various elemental concentrations.

Per Directive (EU) 2018/850, the landfilling of waste containing significant amounts of bio-waste should be limited or forbidden. Due to greenhouse gas emissions, such waste should be managed by digestion or energy conversion, as per Directive 2008/98/EC. Given the above, using diverse methods such as selective digestion, manual and mechanical sorting, C14 carbon isotope determination [40] and optical microscopy, which was applied in this study, is extremely significant in the process of organic fraction content identification in waste. Furthermore, optical tests make it possible to determine the contents of other solids, which can facilitate decision making concerning the further processing or storage of residual waste. As revealed by the microscopic studies of the collected samples, the recycled material from commingled household waste is rich in the bio-waste fraction, at a level from 28.4% for sample no. 5 to 90.7% for sample no. 3. At the same time, as the amount of bio-waste in the samples decreases, an increase in the petroleum products can be observed, from 7% for sample no. 3 to 45.9% for sample no. 5. Similarly, a reduction in the bio-fraction content correlates with an increase in the inorganic matter fraction in the waste. Its content ranges from 2.2% in sample no. 3 to 24.8% in sample no. 5. Slight amounts of the remaining solids, such as hard coal, charcoal and other unidentified substances, present in quantities from 0.1% to 0.6%, have no significant influence on the physicochemical properties of the tested waste. Therefore such low quantities of certain components found in waste will have no impact on decisions concerning the storage, digestion or incineration of waste with similar composition [41,42]. The obtained test material incineration results made it possible to observe the influence of the bio-waste fraction content on the individual incineration process parameters (Table 2-A), along with a graphical representation of the correlations (Fig 3). The increase in organic matter content is accompanied by a clear rise in particulate matter emissions, where in the case of sample no. 3 with the highest bio-waste content, the dust emission was measured at a level of 393.47 mg/m^3^. On the other hand, for sample no. 5 where the lowest organic fraction content was found, the dust emission measurement was nearly 30% as low, at a level of 124.96 mg/m^3^. The authors believe that the determination of this relationship may have a significant effect on optimising the waste disposal process in incineration plants, particularly in the context of bio-component content in the residual fraction. It is especially important as thus far most analyses of emissions from incineration plants neglected this issue [43–45]. A similar increasing emission trend is visible for carbon monoxide in relation to the organic fraction content in the incinerated waste [46,47]. The measuring devices indicated similar CO emissions for samples no. 5 and 4, where the bio-waste content was at a level of 28.4–38.8%. The CO emission exhibited a significant increase when the organic matter content reached 63.6% (sample no. 6), and the emission rate for this sample was noted at a level of 1340.75 mg/m^3^, while for sample no. 3 that had the highest bio-waste content, the emission rate was as high as 2161.67 mg/m^3^ [48]. Another relationship between the bio-waste content and increased emissions was found for VOC, and while no emissions were noted for the incineration of sample no. 5, a clear increase in VOC emissions was revealed for samples no. 7 and 3 with the highest bio-waste fraction content. The remaining measured emission parameters (SO_2_, NOx, O_2_) and technical parameters (power, furnace temperature) exhibited no relationship with the bio-waste content in the individual samples. In the case of the technical parameters, only a decrease in flue gas temperature was observed, inversely proportional to the bio-waste fraction, i.e., the higher the bio-waste content, the lower the flue gas temperature. On the other hand, the positive correlation between the greater organic fraction content in the waste and the increased emissions of particulate matter, carbon monoxide, carbon dioxide and volatile organic compounds, also as described in literature, serves as a reminder that certain researchers also indicate a relationship between the residual fraction composition and sulphur or nitrogen oxides [49–51]. However, no such relationships with regard to the quantitative bio-waste content in the tested material were found in this study. The authors believe that the remaining components of the tested waste have a greater influence on the emissions of these compounds, particularly the petroleum product fraction. Table 2-B, along with a graphical representation of the correlations (Fig 4) presents the relationships and coefficients of correlation between the quantitative bio-waste content and ash, combustion heat, calorific value and calcium oxide, chlorine, sulphur and mercury as found in the test material. It was observed that increased organic component content in the tested waste is related to a gradual increase in calcium oxide and chlorine volumes in samples with high bio-waste content. In samples with the highest organic waste contents (7 and 3), chlorine was found at a level exceeding 0.74%, while calcium oxide exceeded 3.22%. For samples with lower bio-waste contents (5 and 4), the calcium oxide was at a level of 1.78 and 1.95% respectively, while a clear increase in this compound in the waste was found from sample no. 6 onward, which contained over 60% organic matter in its composition. In the case of the measured chlorine values in the individual samples, the reason for the lowest chlorine content in sample no. 4 remains unclear. The authors found no information in the available literature as to which of the remaining components may have led to such a reduction. The fact may be attributed to a laboratory error or the influence of other components. A similar concentration of chlorine in samples no. 5 and 6 is also visible, where the organic fraction content ranges within 38–63%. This suggests that sample no. 4 should also exhibit comparable chlorine content. On the other hand, sulphur content shows an inverse trend, with a decrease as the organic fraction content in the samples increases. A stable sulphur concentration was observed in samples no. 5, 4 and 6, at a level of about 0.19%. However, a sharp decrease in the content of this element, by nearly half, was found in samples no. 7 and 3, to a value of 0.1%. The combustion heat and calorific value analysis results indicate that the highest levels of these parameters were obtained for waste designated as samples no. 4 and 6. In their case, the high values are probably related to the greater petroleum product fraction content (plastics, detergents, cosmetics). In the case of samples no. 7 and 3, the lower combustion heat and calorific value could be associated with the high bio-waste content and humidity of these samples. Meanwhile sample no. 5 was characterised by a high incombustible mineral fraction content (inorganic matter) relative to the calorific fraction of petrochemical origins, which affected the low calorific value of this waste. Table 2-C, along with a graphical representation of the correlations (Fig 5) presents the XRF analysis results for the samples, with 1 ppm accuracy, illustrating the correlation between the individual element content and the percentage bio-waste contribution in the residual waste. An increasing trend for the individual element contents, including aluminium, is observed together with the increase in the organic fraction content in the waste. In the case of aluminium, the increase is proportional and symmetric for samples no. 5, 6 and 7. Slight decreasing variations were noted for sample no. 3, whereas for sample no. 4 a significant reduction in aluminium was observed. The clear decrease in the content of this element in the waste may be related to the proportion of aluminium to the mineral matter in the inorganic fraction as determined by petrographic analysis. Magnesium is another element with an increasing trend analogous to the organic fraction content in the waste. The highest concentration of this element was found in samples no. 7 and 3. Furthermore, in samples with gradually increasing organic matter content, a proportional increase in sulphur, calcium carbonate, chlorine, potassium and phosphorus volumes can be observed as well. The remaining identified elements and chemical compounds do not exhibit clear cumulative tendencies depending on the bio-waste fraction increase in the waste. However, a special relationship was observed in the case of the low uranium volumes, where the accumulation of this element decreased gradually together with the increase in the bio-waste contribution in the samples.

**Table 2:** Determined correlations between organic matter and individual parameters in residual fraction samples. A – individual sample incineration parameter analysis relative to the bio-waste fraction content in the individual samples. B – physicochemical parameter analysis relative to the bio-waste fraction content in the individual samples. C – elemental concentration analysis relative to the bio-waste fraction content in the individual samples.

A										
Variable: Organic matter	The determined coefficients of correlation are relevant at p <.05000 N=5									
	mg/m^3^	mg/m^3^	mg/m^3^	mg/m^3^	mg/m^3^	mg/m^3^	%	°C	kW	°C
	Dust	SO_2_	NOx	CO	CO_2_	VOC	O_2_	Ts	Power	Tp
.9675	.5342	-.4001	.9526	.7549	.9067	-.5470	-.8554	.3471	-.0732
p=.007	p=.354	p=.505	p=.012	p=.140	p=.034	p=.340	p=.065	p=.567	p=.907
**B**										
Variable: Organic matter	The determined coefficients of correlation are relevant at p <.05000 N=5									
	%	%	J/g	J/g	%	%	%	mg/kg		
	Humidity	Ash	∆Hc	CV	S	CaO	Cl	Hg		
	.7863	-.5825	-.4948	-.5498	-.9126	.9649	.8858	.2904		
	p=.115	p=.303	p=.397	p=.337	p=.031	p=.008	p=.046	p=.636		
**C**										
Variable: Organic matter	The determined coefficients of correlation are relevant at p <.05000 N=5									
	ppm	ppm	ppm	ppm	ppm	ppm	ppm	ppm	ppm	ppm
	Al	As	Ba	Cd	Cu	Fe_2_O_3_	K	La	Mg	Mn
	.9148	-.1322	.4477	-.2647	-.4127	.9454	.7851	.7616	.9033	.7641
	p=.029	p=.832	p=.450	p=.667	p=.490	p=.015	p=.116	p=.135	p=.036	p=.133
	ppm	ppm	ppm	ppm	ppm	ppm	ppm	ppm	ppm	ppm
	Mo	Nd	Ni	P	Pb	Pr	Si	Ti	U	Zn
	-.4080	.9058	.6333	.9264	-.0919	.6625	.3336	.4925	-.9734	.0586
	p=.495	p=.034	p=.251	p=.024	p=.883	p=.223	p=.583	p=.399	p=.005	p=.925

**Fig 6:**
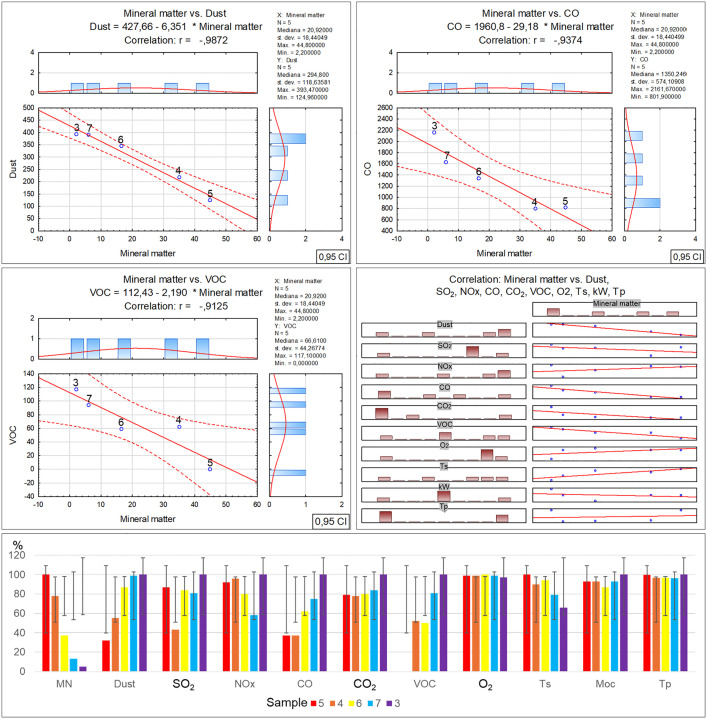
Correlation and regression analysis between inorganic matter content and the parameters detailed in Table 3A for residual waste fraction samples.

Analysing the results obtained during the sample incineration revealed that in the case of dust emission measurements an inverse relationship was observed between the inorganic matter content and the dust emission compared to the relatively higher organic matter content. A greater contribution of inorganic matter in the waste led to a lower dust emission (Table 3-A), along with a graphical representation of the correlations (Fig 6). A similar relationship can also be noted in the case of CO emissions, where a clear increase in emission is observable from sample no. 6, which contained 16.6% of inorganic matter, to the maximum CO emission for sample no. 3 with the lowest inorganic fraction content of 2.2%. On the other hand, samples with inorganic matter content of 35% (sample no. 4) to 44.8% (sample no. 5) exhibited an emission of this compound at a low level of about 800 ppm. A slight increase in CO_2_ emissions was observed as well in samples with a lower proportion of the inorganic fraction to the organic, with a clear maximum value for sample no. 3. Volatile organic compound (VOC) emission measurements demonstrated that the emissions of these compounds were also inversely proportional to the inorganic matter content in the samples [52]. It should be noted that the above analyses do not exhibit a full correlation with studies presenting the results of emission measurements as described in literature, as some of them state that, e.g., a greater contribution of mineral matter in the waste leads to greater emissions of dust, CO and CO_2_ [53,54]. However, while the percentage contribution of the mineral fraction in household and residual waste is provided in literature, the specific composition of this fraction remains undefined in these publications. The lack of a detailed analysis of the mineral fraction composition constitutes a significant limitation in assessing the potential for waste incineration in low-power heating devices (in temperatures of up to 700°C), particularly in the context of materials such as glass, slag and ceramics, which do not undergo thermochemical conversion, and whose presence thereby leads to the enrichment of ash rather than an increase in emissions. Among the recorded technical parameters, the only change was found in a slight flue gas temperature decrease, proportional to the decreasing inorganic matter content in the samples. The remaining incineration parameters, such as the power and furnace temperature, remained stable regardless of the different contents of this component in the tested waste.

**Table 3:** Determined correlations between inorganic matter and individual parameters in residual fraction samples. A – individual sample incineration parameter analysis relative to the inorganic matter content. B – physicochemical parameter analysis relative to the inorganic matter content. C – elemental concentration analysis relative to the inorganic matter content in the individual samples.

A										
Variable: Inorganic matter	The determined coefficients of correlation are relevant at p <.05000 N=5									
	mg/m^3^	mg/m^3^	mg/m^3^	mg/m^3^	mg/m^3^	mg/m^3^	%	°C	kW	°C
	Dust	SO_2_	NOx	CO	CO_2_	VOC	O_2_	Ts	Power	Tp
-.9872	-.4983	.3952	-.9374	-.7212	-.9125	.5070	.8279	-.2761	.1359
p=.002	p=.393	p=.510	p=.019	p=.169	p=.031	p=.383	p=.083	p=.653	p=.827
**B**										
Variable: Inorganic matter	The determined coefficients of correlation are relevant at p <.05000 N=5									
	%	%	J/g	J/g	%	%	%	mg/kg		
	Humidity	Ash	∆Hc	CV	S	CaO	Cl	Hg		
	-.7340	.6573	.4107	.4689	.8677	-.9792	-.8351	-.3191		
	p=.158	p=.228	p=.492	p=.426	p=.057	p=.004	p=.078	p=.601		
**C**										
Variable: Inorganic matter	The determined coefficients of correlation are relevant at p <.05000 N=5									
	ppm	ppm	ppm	ppm	ppm	ppm	ppm	ppm	ppm	ppm
	Al	As	Ba	Cd	Cu	Fe_2_O_3_	K	La	Mg	Mn
	-.8715	.1592	-.4700	.1632	.3710	-.9063	-.7668	-.7589	-.8616	-.7059
	p=.054	p=.798	p=.424	p=.793	p=.539	p=.034	p=.130	p=.137	p=.060	p=.183
	ppm	ppm	ppm	ppm	ppm	ppm	ppm	ppm	ppm	ppm
	Mo	Nd	Ni	P	Pb	Pr	Si	Ti	U	Zn
	.4259	-.8932	-.5537	-.8875	.0778	-.6678	-.3052	-.5096	.9810	-.1353
	p=.475	p=.041	p=.333	p=.045	p=.901	p=.218	p=.618	p=.380	p=.003	p=.828

**Fig 7:**
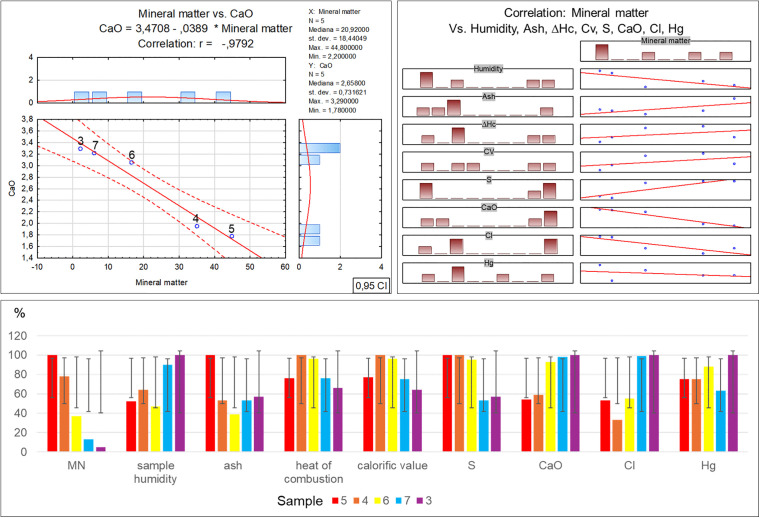
Correlation and regression analysis between inorganic matter content and the parameters detailed in Table 3B for residual waste fraction samples.

A marked increase in ash, related to the inorganic matter proportion, is clearly observable in sample no. 5, which exhibits the highest ash content (29.6%). The ash content in the remaining samples is at a similar level of 11.6% to 16.8%, with no clear increasing trend depending on the percentage contribution of inorganic matter in the waste. The determined elemental contents in the individual samples show a clear decrease in sulphur content in samples no. 7 and 3, which contained the least inorganic matter. However an inverse trend was observed for chlorine in samples 7 and 3, where the highest concentration of this element was found. As regards the calcium oxide concentration, a successive increase in this compound was noted in samples no. 5 and 4, and afterwards in samples 6, 7 and 3, with the simultaneous percentage reduction in the inorganic matter content in the waste (Table 3-B), along with a graphical representation of the correlations (Fig 7).

**Fig 8:**
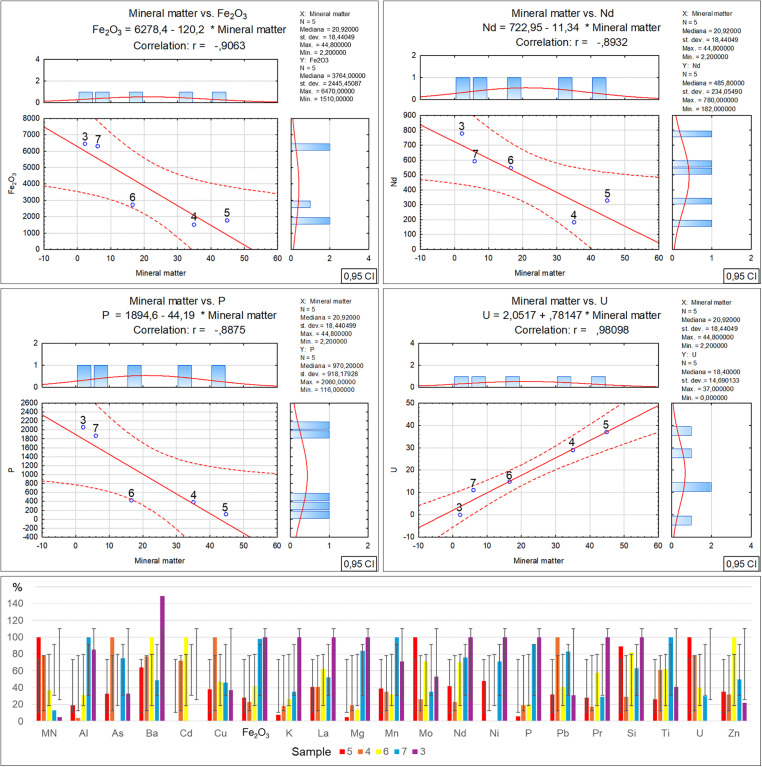
Correlation and regression analysis between inorganic matter content and the parameters detailed in Table 3C for residual waste fraction samples.

Analysing the elemental content of the tested samples revealed significant relationships between the inorganic matter fraction content and the concentrations of selected elements. It was found that the iron oxide, neodymium, potassium and phosphorus concentrations exhibit an inverse proportionality to the quantity of inorganic matter in the tested waste, and that they decrease together with the increase in the inorganic fraction. On the other hand, the aluminium and barium concentrations change in an irregular manner, but also with a rising tendency together with the decrease in the inorganic matter content. Meanwhile the uranium concentration increases proportionally to the percentage inorganic fraction contribution in the waste (Table 3-C), along with a graphical representation of the correlations (Fig 8). Literature data concerning the correlations between the inorganic matter content and the presence of certain elements and chemical compounds [55,56] indicates the potential efficiency of the waste recycling processes as well as the component origins. The presence of the elements and chemical compounds presented above as well as their concentrations in the residual waste suggests that the recovery of metals and other inorganic fractions was performed with high efficiency.

**Fig 9:**
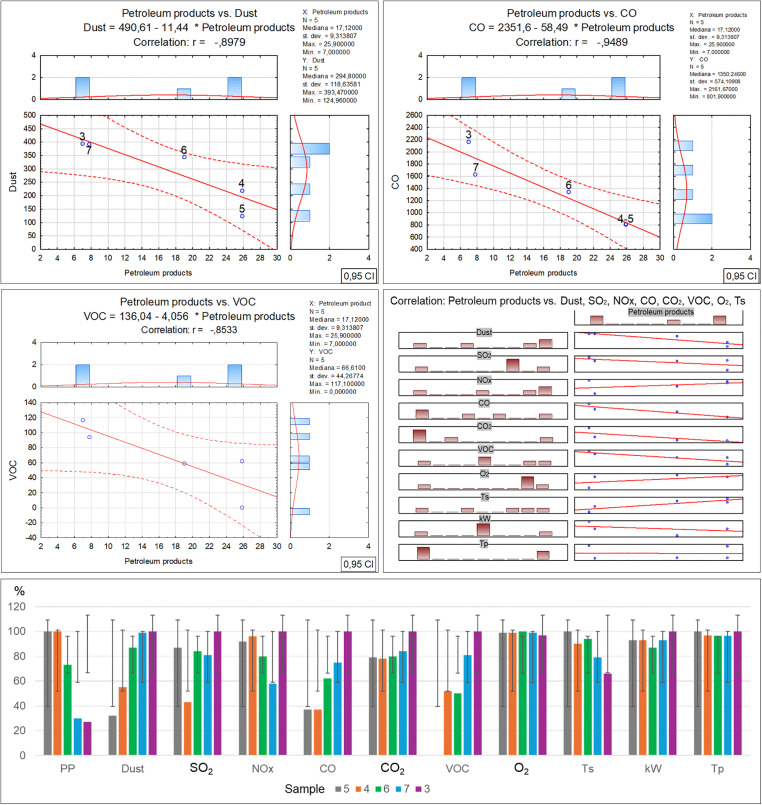
This figure illustrates the statistical relationship between Petroleum Products and environmental parameters in Table 4 A.

Table 4 and Fig 9 show how the percentage of petroleum product fractions influences incineration processes, physicochemical properties, and elemental content in waste containing up to 30% petroleum products. The result analysis revealed complex relationships that are not immediately obvious based on a review of prior studies described in literature [57,58]. Particularly clear correlations were observed in the recorded parameters of dust, carbon oxide, carbon monoxide and volatile organic compound emissions. A distinct trend indicates that the minimum petroleum product fraction content in the mixture results in the maximum emissions of these gases and particulate matter due to the increase in the organic fraction content. On the other hand, a higher proportion of petroleum products affects the flue gas temperature increase and NOx emission fluctuations.

**Table 4:** Determined correlations between petroleum products and individual parameters in residual fraction samples. A – individual sample incineration parameter analysis relative to the petroleum product content in the individual samples. B – physicochemical parameter analysis relative to the petroleum product content in the individual samples. C – elemental concentration analysis relative to the petroleum product content in the individual samples.

A										
Variable: Petroleum products	The determined coefficients of correlation are relevant at p <.05000 N=5									
	mg/m^3^	mg/m^3^	mg/m^3^	mg/m^3^	mg/m^3^	mg/m^3^	%	°C	kW	°C
	Dust	SO_2_	NOx	CO	CO_2_	VOC	O_2_	Ts	Power	Tp
-.8979	-.6015	.4176	-.9489	-.7844	-.8533	.5900	.8669	-.4555	-.0467
p=.039	p=.283	p=.484	p=.014	p=.116	p=.066	p=.295	p=.057	p=.441	p=.941
**B**										
Variable: Petroleum products	The determined coefficients of correlation are relevant at p <.05000 N=5									
	%	%	J/g	J/g	%	%	%	mg/kg		
	Humidity	Ash	∆Hc	CV	S	CaO	Cl	Hg		
	-.8511	.4117	.6463	.6921	.9708	-9107	-.9609	-.2128		
	p=.067	p=.491	p=.239	p=.195	p=.006	p=.032	p=.009	p=.731		
**C**										
Variable: Petroleum products	The determined coefficients of correlation are relevant at p <.05000 N=5									
	ppm	ppm	ppm	ppm	ppm	ppm	ppm	ppm	ppm	ppm
	Al	As	Ba	Cd	Cu	Fe_2_O_3_	K	La	Mg	Mn
	-.9747	.0918	-.3745	.4567	.5009	-.9916	-.7818	-.7334	-.9483	-.8570
	p=.005	p=.883	p=.535	p=.439	p=.390	p=.001	p=.118	p=.159	p=.014	p=.064
	ppm	ppm	ppm	ppm	ppm	ppm	ppm	ppm	ppm	ppm
	Mo	Nd	Ni	P	Pb	Pr	Si	Ti	U	Zn
	.3416	-.9061	-.7670	-.9675	.1272	-.6246	-.3906	-.4498	.9230	.0732
	p=.574	p=.034	p=.130	p=.007	p=.839	p=.260	p=.516	p=.447	p=.025	p=.907

**Fig 10:**
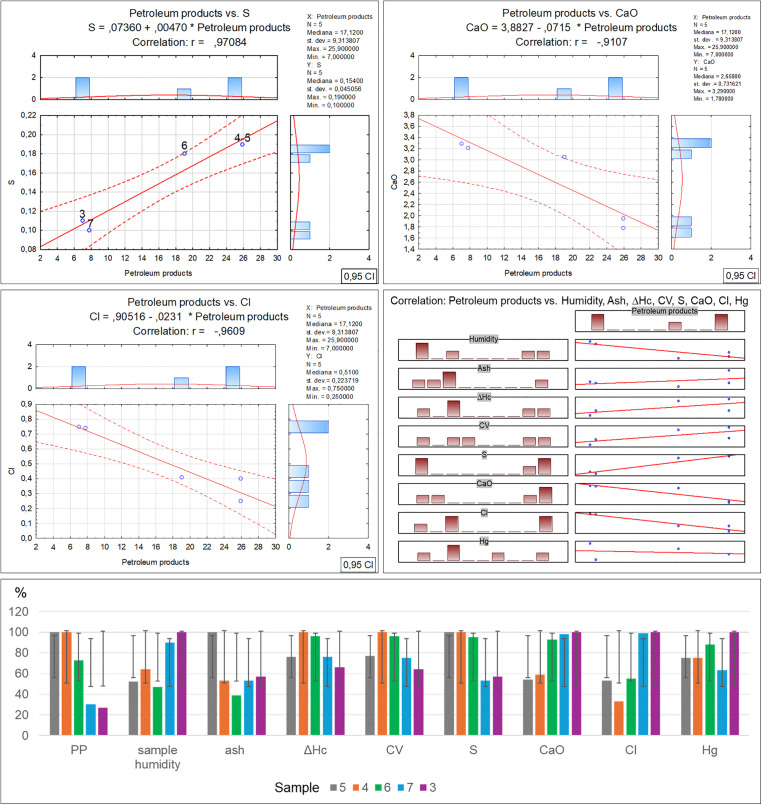
This figure illustrates the statistical relationship between Petroleum Products and environmental parameters in Table 4 B.

Further result analysis demonstrated (Table 4-B and Fig 10) that the flue gas temperature and combustion heat increase proportionally to the petroleum product content in the tested waste, with one exception in sample no. 5. The decrease of these parameters in this sample, despite a similar petroleum product content (about 30%) as in sample no. 4, can be attributed to the 9.8% higher inorganic matter content, which does not participate directly in low-temperature exothermic processes. The higher petroleum product content leads to a successive increase in sulphur concentration in the tested samples, but with a clear inverse trend in the case of calcium oxide and chlorine.

**Fig 11:**
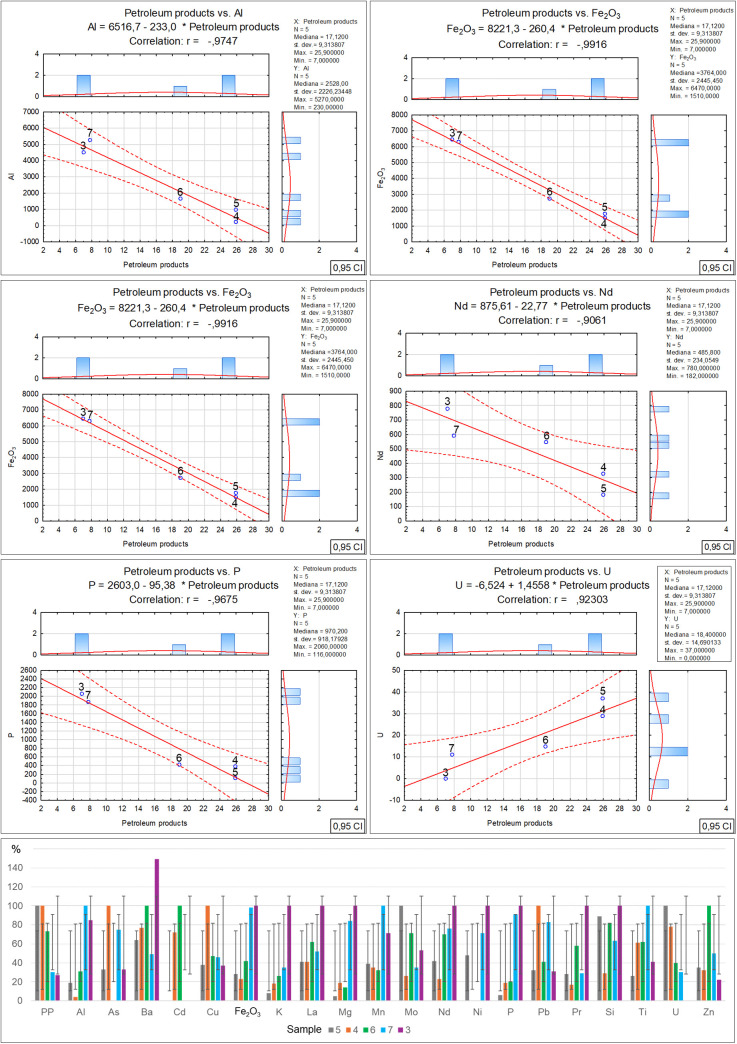
This figure illustrates the statistical relationship between Petroleum Products and environmental parameters in Table 4 C. kW – power; Ts – flue gas temperature; Tp – furnace; temperature; ∆Hc – combustion heat.

The identified elemental contents (Table 4-A and Fig 11) in the individual samples are significant in the case of aluminium, magnesium, niobium, phosphorus and iron oxide, revealing a distinct reduction in the concentrations of these elements and compounds that is parallel to the decrease in the petroleum product contents in the samples. A variable heavy metal content (As, Cd, Nd, Hg) in the individual samples can also be observed, though in this case the concentrations of these elements cannot be directly associated with the percentage petroleum product content in the tested samples.

## 5. Conclusions

The conducted tests were intended to determine the solid components present in residues remaining after household waste recycling processes. A significant element of the analysis was to inspect the influence of these components on the incineration processes, physicochemical properties and elemental concentrations in the analysed residual fraction. The quantity and quality of the residual fraction components was determined not only through chemical methods, but also by a novel method of microscopic analysis. The method enables the precise quantitative and qualitative determination of components such as: animal-based waste, cellulose, modern and woody biomass, wood tar, coal, coke, plastics, rubber, paint, grease, glue, polymer resin, tar, liquids, sand, quartz, soil, stone powder, ceramics, glass, metal, rust, ash, slag and others. The component identification for the purposes of this study was limited to determining the percentage contribution of the basic groups forming the waste. These groups included: organic matter, inorganic matter, petroleum products as well as other characteristic components that cannot be assigned to these basic categories. The analyses revealed that these groups have great significance for downstream processes such as incineration, storage and landfilling, or other decision making, e.g., concerning the further recycling of the waste. The presented relationships between the waste composition and processing are very important for optimising the incineration processes, minimising pollutant emissions and assessing the environmental risks related to the waste-to-energy conversion conducted in low-power facilities. Analysing the test results revealed that waste with high organic matter content has the greatest influence on the increase in particulate matter, carbon oxide, carbon monoxide and volatile organic compound emissions. It was observed that the biomass content in the samples increased threefold from the minimum to the maximum value, which correlated with a threefold increase in particulate matter emissions and a 2.5-fold increase in carbon monoxide emissions. This phenomenon is related to the diversity and type of biomass, as the organic matter in household waste originates from both animal and plant sources. Furthermore, the advanced decomposition level of the organic fraction likely affects the increased emissions during the incineration of waste with high organic matter content. Additionally, in samples with increasing biomass content, a significant increase in moisture (from 14% to 27%) was observed, which also affected the combustion conditions and exhaust gas quality. It should be noted that due to the lack of a material for comparison, the paper does not determine which of the organic fraction components (plant- or animal-based) exhibits the dominant influence on the pollutant emissions during waste incineration. This issue requires further research encompassing the optical analysis of the samples, with a focus on determining the origins of the organic matter in the waste and simulating an incineration process using materials with a similar general composition of the organic matter but different origins of the individual components. Nevertheless, the basic analysis of the organic matter proportion to the other component groups is already relevant, e.g., for developing a strategy for residual waste management, factoring in the solid fraction composition analysis results, which would enable an optimisation of the further directions for residual waste repurposing.
